# Ionic liquids as transesterification catalysts: applications for the synthesis of linear and cyclic organic carbonates

**DOI:** 10.3762/bjoc.12.181

**Published:** 2016-08-26

**Authors:** Maurizio Selva, Alvise Perosa, Sandro Guidi, Lisa Cattelan

**Affiliations:** 1 Dipartimento di Scienze Molecolari e Nanosistemi, Università Ca’ Foscari Venezia, Via Torino, 155 – Venezia Mestre, Italy

**Keywords:** ionic liquids, transesterification, organocatalysts, organic carbonates

## Abstract

The use of ionic liquids (ILs) as organocatalysts is reviewed for transesterification reactions, specifically for the conversion of nontoxic compounds such as dialkyl carbonates to both linear mono-transesterification products or alkylene carbonates. An introductory survey compares pros and cons of classic catalysts based on both acidic and basic systems, to ionic liquids. Then, innovative green syntheses of task-specific ILs and their representative applications are introduced to detail the efficiency and highly selective outcome of ILs-catalyzed transesterification reactions. A mechanistic hypothesis is discussed by the concept of cooperative catalysis based on the dual (electrophilic/nucleophilic) activation of reactants.

## Review

### Introduction

#### Transesterification catalysts

**Scheme 1:**
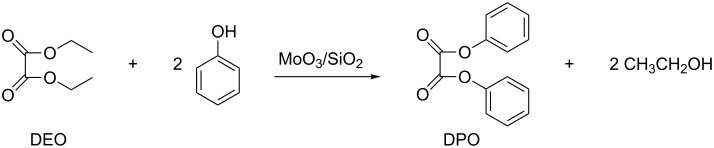
The transesterification of diethyl oxalate (DEO) with phenol catalyzed by MoO_3_/SiO_2_.

The transesterification is one of the classical organic reactions that has found numerous applications in laboratory practice as well as in the synthesis of a variety of intermediates in the pharmaceutical, cosmetic, fragrance, fuel and polymers industries [1]. Transesterification reactions are catalyzed under acidic, basic or even neutral conditions [2]. An excellent review by Otera et al. has detailed many applications of the most popular catalytic systems [3]. These include both acids such as sulfuric, sulfonic, phosphoric, and hydrochloric, and bases such as metal alkoxides, acetates, oxides, and carbonates. It is worth mentioning, that transesterification reactions are frequently carried out over solid (heterogeneous) catalysts to facilitate work-up, recycling, and purification of products, especially for large-scale preparations. These heterogeneous systems include supported metal oxides and binary oxide mixtures. For example, MoO_3_/SiO_2_ and sol–gel MoO_3_/TiO_2_ is used for the preparation of diphenyl oxalate monomer (DPO, Scheme 1) in polycarbonate chemistry [4–5], and TiO_2_/SiO_2_ and similar binary combinations are applied in the transesterification of β-ketoesters [6], and in the synthesis of unsymmetrical carbonates R^1^OC(O)OR^2^ [7].

Superacidic solids have also been described as transesterification catalysts and a remarkable example is the recently patented synthesis of sucrose-6-ester – a food sweetener – carried out over a mixture of sulfated oxides of various metals [8]. In addition, acidic ion exchange resins are worth mentioning in this context. Van de Steene et al. have proved the performance of such systems in an elegant investigation on the model transesterification of ethyl acetate with methanol [9].

**Scheme 2:**
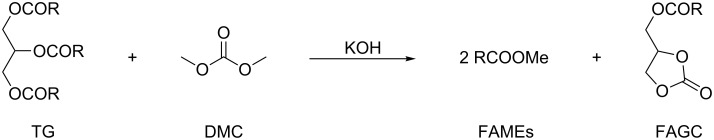
Transesterification of a triglyceride (TG) with DMC for biodiesel production using KOH as the base catalyst.

The production of biodiesel blends is another sector in which the catalytic transesterification is extensively used. In particular, heterogeneous catalysts including calcium, manganese and zinc oxides as such or as mixtures are widely used to convert natural triglycerides into FAMEs or FAEEs (fatty acid methyl or ethyl esters) with methanol or ethanol, respectively [10]. The most commonly used system is CaO, which is obtained by calcination of readily available and cheap resources including waste products such as shells and even livestock bones [11–14]. However, traditional catalysts such as alkali hydroxides or alkaline methoxides are still encountered even for novel syntheses of biofuels. An example is the transesterification of oils by dimethyl carbonate (DMC) in the presence of KOH (Scheme 2) [15–16].

The reaction allows obtaining FAMEs and fatty acid glycerol carbonate monoesters (FAGCs), without the concurrent formation of glycerol, a frequently formed highly undesirable byproduct.

**Enzyme catalysts:** A major driving force for the choice of enzymes is their high efficiency, which allows reactions to be performed under very mild conditions and with a variety of raw materials. However, the high cost and relatively short lifetime of enzymes partly offset their advantages and an implementation of biocatalytic processes makes sense almost exclusively for the preparation of high added-value chemicals. This holds true also for enzyme-catalyzed transesterification reactions. To cite a few examples, the literature claims the use of lipase as a biocatalyst for i) the reaction of glycerol with DMC for the synthesis of glycerol carbonate (GlyC) under solvent-free conditions. A 60% yield was achieved along with an effective recycle of the catalyst [17], ii) the formation of six-membered cyclic carbonates by the transesterification of dialkyl carbonates with trimethylolpropane. The products were achieved in high yields (85%) and used as monomers for polyurethanes and polycarbonates [18], and iii) the conversion of oils for which lipase was identified as the most suitable enzyme for an innovative and green production of biodiesel [19].

**Other catalytic systems:** In addition to the above-described catalysts, amines and organometallic derivatives also find applications in the field of homogeneous catalytic systems for transesterification reactions. Remarkable examples are those of triethylamine (TEA) and Fe–Zn double-metal cyanide complexes [20–21]. Among other applications, these compounds successfully catalyzed the reaction of DMC and other organic carbonates with polyols (e.g., glycerol) to produce the expected transesterification products with total conversion and selectivity.

### Ionic liquid-based organocatalysts

Conventional acid or base liquid catalysts for transesterification processes often entail several synthetic and environmental concerns including equipment corrosion, separation and purification drawbacks, and production of waste. As already mentioned in the previous paragraph, practical solutions to such problems are offered by using solid acids, although these systems may suffer from mass-transfer limitations causing low activity, and consequently, extended reaction times and deactivation from coking [22–23]. Valuable alternatives are biocatalysts, which are very active but costly. Economic issues usually restrict the use of enzymes to highly specialized productions rather than to large commercial applications [24].

In this scenario, the implementation of transesterification procedures based on innovative and possibly green catalysts remains still a highly desirable target. A strategy can be conceived by the use of task-specific ionic liquids (ILs). These compounds have shown to catalyze a number of different reactions. Only to cite a few: nitrations, Michael reactions, Friedel–Crafts alkylations and acylations are successfully promoted by ILs [25–26]. The key to such a flourishing research lies in the unique physical properties (negligible vapor pressure, wide liquid range, and non-flammability) of ILs, but mostly on the virtually infinite number of different chemical structures for liquid organic salts. These properties are often referred to as “tunable catalysts”, “task-specific ionic liquids”, and “designer solvents”, which involve the concept of optimizing the use of ILs by tailoring their chemical features for a specific transformation or for classes of similar processes [27–28]. Notably, the screening of the reaction variables includes not only the required reaction steps, but also the associated operations including separation and purification of products, recycling of solvents and catalysts, and waste treatments as well. All these additional steps contribute to the impact of the chemical process as the whole from an environmental and sustainability standpoint. For example, the isolation and purification of the desired product and reuse of the IL-based catalyst may require additional solvents for extraction and/or complex and energy-intensive separation and purification technologies. Therefore, when designing a catalytic IL-based process, one should factor-in all the reagents and solvents as well as all the downstream operations, in order to evaluate the advantages of the proposed process correctly. In this context, green metrics can provide a screening guide.

#### IL-based catalysts for transesterification reactions

**Synthesis of IL-catalysts:** IL-based catalysts for transesterification reactions mostly comprise imidazolium, phosphonium, ammonium, sulfonium and pyridinium salts. The conventional syntheses of such compounds usually start from the protonation or quaternization of neutral precursors (imidazoles, amines, phosphines, pyridine or sulfides) with Brønsted acids or haloalkanes/dialkylsulfates, respectively. In the next step, a variety of ionic liquids are obtainable by anion exchange, either through direct treatments with Lewis acids or by anion metathesis [29]. There are several reviews detailing these synthetic procedures [30–31].

**Scheme 3:**
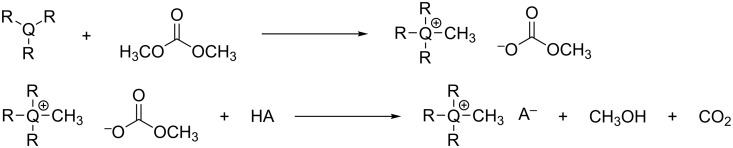
Top: Green methylation of phosphines and amines by dimethyl carbonate (Q = N, P). Bottom: anion metathesis of methyl carbonate onium salts.

More sustainable methods that avoid the use of noxious and undesirable halogens have also been recently designed [32–33]. An example is the preparation of methyl carbonate onium salts ([Q_1_*_nnn_*][MeOCO_2_]; Q = N, P; *n* = 4, 6, 8, Ph), obtained by the methylation of trialkylphosphines or -amines with nontoxic DMC (Scheme 3, top) [34–35]. Such methyl carbonate onium salts are versatile platforms as they allow access to a number of ionic liquids via anion-metathesis reactions, which produce only CH_3_OH and CO_2_ as byproducts (Scheme 3, bottom).

Seedon et al. reported another green protocol for the preparation of ILs. The authors described the synthesis of aqueous hydroxide solutions of organic cations, subsequently neutralized by simple acid–base reactions, giving access to ionic liquids that are difficult to prepare by any other route. This protocol avoids the use of halides, and generates water as the only byproduct [33].

**Synthesis of supported ionic liquids (SILs):** Ionic liquids are far more expensive than classical solvents, with costs higher by a factor of 10-to-50. The recycling of the ILs is therefore imperative not only to limit their release to the environment, but also for economic reasons. One strategy to cope with the recycling issue is based on the immobilization of ionic liquids onto solid supports. In the specific field of transesterification reactions, supported ionic liquid (SILs) catalysts are achieved by the dispersion of liquid organic salts on highly porous materials, amongst which montmorillonite clays, modified silica, and polystyrene-based solids are the most frequently used [36–37]. Some very recent examples described the production of biodiesel via the transesterification of glycerol trioleate with methanol: both, acidic ionic liquids (e.g., 1-allyl-3-(butyl-4-sulfonyl)imidazolium trifluoromethanesulfonate [BsAIm][OTf]) supported onto sulfhydryl-group-modified SiO_2_ (MPS-SiO_2_) [38], and imidazolium salts (e.g., 1-allyl-dodecylimidazolium hydroxide ([ADIm][OH]) dispersed on magnetic mesoporous SiO_2_/CoFe_2_O_4_ and CoFe_2_O_4_ nanoparticles [39–40] have been reported as catalysts. In addition, the reaction of ethylene carbonate with methanol for the synthesis of DMC was described in the presence of a mesocellular silica foam (MCF) material [41]. These catalysts are easy to recover and recycled by physical separation, washing and drying.

**Figure 1:**
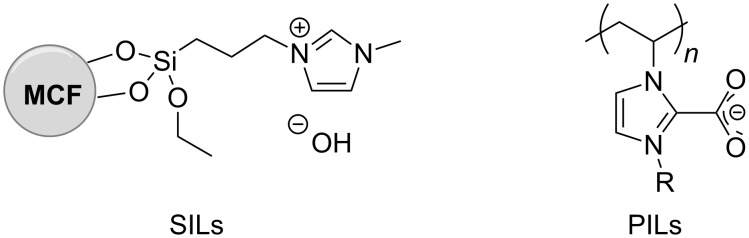
Structures of some representative SILs and PILs systems. MCF is a silica-based mesostructured material with ultra-large mesopores of 20–50 nm [42–43].

A similar approach has been implemented through the design of polymeric ionic liquid (PILs) based systems, such as poly(*N*-heterocyclic carbene)s and ordered mesoporous resol (OMR) polymers (OMR based on hexamethylenetetramine, [C_4_HMTA][SO_4_H]). They have been employed to catalyze different transesterification reactions, including also the conversion of brown grease into biodiesel [42–43]. Recycling tests of polymeric ionic liquids proved their robustness for prolonged use (Figure 1).

**Scheme 4:**
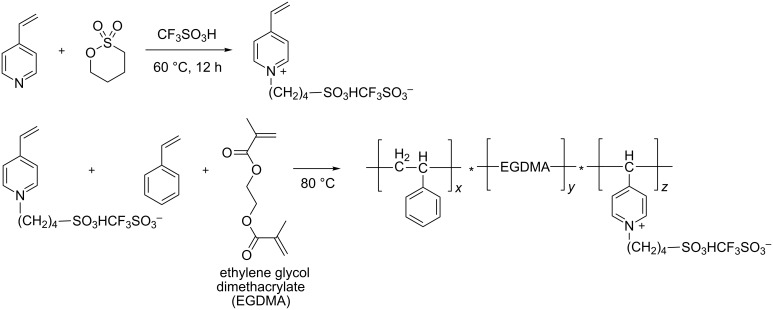
Synthesis of the acid polymeric IL. EGDMA: ethylene glycol dimethacrylate.

Recently, Zhan et al. synthesized a new acidic polyionic liquid by the copolymerization of a zwitterionic liquid based on vinylpyridinium, styrene and ethyleneglycol dimethacrylate (Scheme 4) [44]. The resulting PIL with particle sizes of about 0.5–3 mm, was an efficient catalyst for a series of esterification reactions of different acids including acetic, succinic, benzoic, and methacrylic acid and alcohols such as linear, branched and cyclic C_1_–C_6_ compounds. The PIL could be reused up to five times without any loss of catalytic activity and yields of various esters were always nearly quantitative.

**Applications of ILs:** Organocatalysts find uses in place of the common homogeneous or heterogeneous catalysts for the transesterification of natural triglycerides in the production of biodiesel. A recent example has reported that a methylimidazolium salt with an alkyl chain mimicking the glycerol structure, promotes the almost quantitative conversion of rapeseed oils into FAMEs products [45].

**Scheme 5:**
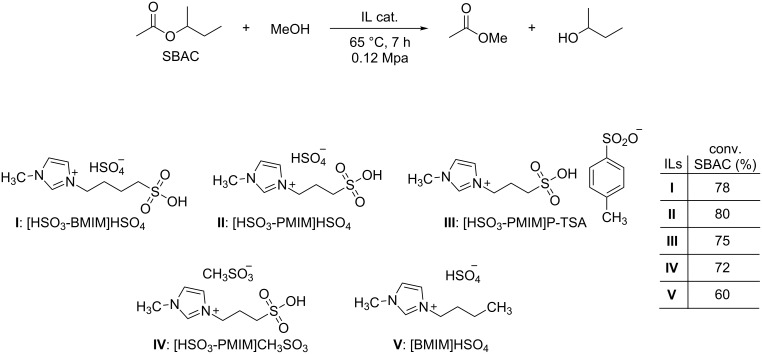
The transesterification of sec-butyl acetate with MeOH catalyzed by some acidic imidazolium ILs.

A series of Brønsted acidic imidazolium ILs has been investigated for the catalytic synthesis of *sec*-butanol by transesterification of *sec*-butyl acetate with methanol (Scheme 5) [46].

The reaction is of interest for the preparation of less toxic oxygenated derivatives, such as *sec*-butanol, in place of compounds like MTBE (methyl *tert*-butyl ether) for the formulation of gasoline blends. Tests with the imidazolium salts collected in Scheme 5 have demonstrated that they are not only competitive with conventional acid catalysts, but that they also can be recovered and reused to allow quantitative conversions even after several recycles. Moreover, the study highlighted that the catalytic activity increased with increasing acidity of the ILs and particularly with cations bearing SO_3_H anions (Scheme 5: ILs **I**, **II**, **III**, and **IV**). The same imidazolium salts (**I** and **II**, respectively) have been used also by Cui et al. for the transesterification of methyl acetate and *n-*butanol [47]. The authors observed that the presence of two acidic sites in both the cation and the anion of ILs improved the performance of the catalyst, in analogy to previously reported results for the synthesis of esters from the reaction of nitriles and alcohols [48].

4-(3-Methyl-1-imidazolium)-1-butanesulfonic acid triflate ([HSO_3_-BMIM][CF_3_SO_3_]) has been chosen as a model organocatalyst to explore the kinetics of the transesterification of methyl acetate with ethanol [49–50]. Again, in this case, the investigation proved that the activity of the organic salt was higher than that of sulfuric acid.

**Figure 2:**
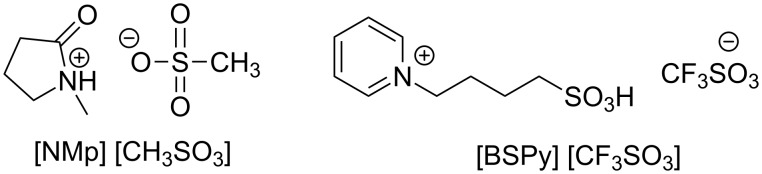
Representative examples of ionic liquids for biodiesel production.

The use of ionic liquids for the catalytic production of biodiesel was recently reviewed by Fauzi and Amin [51], who focused on the improvements made possible by organocatalysts with respect to traditional homogenous systems in terms of milder reaction conditions and easier separation and recycle workups. Two representative examples of ionic liquids employed for the synthesis of FAMEs are the pyridinium and oxazolidinone-based compounds shown in Figure 2 [52–53].

It should be noted that for such reactions, acidic IL-based catalysts are preferred over basic ones due to the presence of significant amounts of free fatty acids in the bio-oils used as feedstocks for biodiesel. Li et al. for example designed an innovative combination of imidazolium ILs and metal sulfates acting as Brønsted and Lewis acids, respectively [54]. A model case is [HSO_3_-BMIM]HSO_4_–Fe_2_(SO_4_)_3_ that offered an excellent catalytic performance in the transesterification of *Camptotheca acuminata* seed oil with methanol, with substantially quantitative conversions achieved in only 60 minutes at 60 °C.

#### The transesterification reaction for the synthesis of organic carbonates

**Scheme 6:**
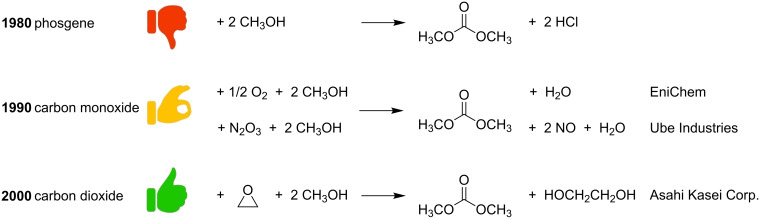
Top: phosgenation of methanol; middle: EniChem and Ube processes; bottom: Asahi process for the production of DMC.

Organic carbonates (OCs) are promising candidates as *green* replacements of conventional noxious solvents and fuel additives as well as for the development of innovative intermediates in the pharma, lubricant and polymer industries [55–56]. Before the 1980’s, the industrial synthesis of the simplest representative of the series, dimethyl carbonate (DMC), was based on the phosgenation of methanol, which used a lethal chemical reagent such as phosgene (Scheme 6, top). Since then, the processes for the production of DMC have progressively evolved in terms of environmental impact, safety and economics.

Thus, by the end of the 1990’s, two main phosgene-free large-capacity processes were operative, both based on the incorporation of carbon monoxide (CO) and methanol by transition metal catalysis: one developed by EniChem [57–58], and the other by Ube Industries [59]. The EniChem process involved an oxidative carbonylation of methanol, i.e., the reaction of methanol with carbon monoxide and oxygen catalyzed by cuprous chloride, while the Ube process used an oxidative carbonylation of methanol via methyl nitrite using NO*x* as oxidant, instead of oxygen and a palladium catalyst (Scheme 6, middle). Though safer than the phosgenation of methanol, these synthetic routes still involved poisonous carbon monoxide and methyl nitrite, and chlorine-based catalysts.

Carbon dioxide is the natural green alternative carbonyl source to these undesirable feedstocks, in particular to CO, except that its thermodynamic stability poses severe challenges. This potential limitation was overcome by the Asahi Kasei Corp. that recently industrialized a catalytic polycarbonate production process based on the use of carbon dioxide (CO_2_) for the synthesis of DMC as an intermediate towards the diphenyl carbonate monomer. The first step is the insertion of CO_2_ into ethylene oxide to give ethylene carbonate, which is catalyzed by onium salts. The second step involves the transesterification of ethylene carbonate with methanol. The reaction is carried out in a continuous distillation reactor loaded with quaternary ammonium strongly basic anion exchange resin and alkali hydroxides: dimethyl carbonate (DMC) is achieved in practically quantitative yields (Scheme 6, bottom). The third and final step is the transesterification of DMC with phenol by a catalytic reactive distillation in the presence of a homogeneous Ti, Bu–Sn, or Pb catalyst. This reaction provides the desired diphenyl carbonate (yield up to 99%) in a high purity [60].

**Scheme 7:**
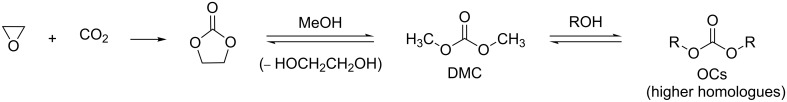
The transesterification in the synthesis of organic carbonates.

The catalytic transesterification appears therefore as a crucial reaction, not only for the preparation of the simplest homologue, dimethyl carbonate (DMC), but also for the synthesis of higher organic carbonates as well (Scheme 7).

Notwithstanding the excellent results with respect to previous methods, it should be pointed out, that ethylene oxide still represents a concern for its carcinogenic and mutagenic properties. Future procedures should therefore implement greener reactions, such as the direct carboxylation of diols by CO_2_, for which however, effective catalysts are currently not available.

The Asahi Kasei process also highlights that the synthesis of DMC by transesterification of ethylene carbonate with methanol does not necessarily require transition metal catalysis as did the EniChem and Ube processes. Instead, the reaction can be effectively catalyzed by a combination of supported basic ammonium resins and homogeneous alkaline bases [60], thereby demonstrating the potential of transition metal-free catalytic systems for the synthesis and the further transformation of organic carbonates. These transesterification catalysts can include both acidic and basic ionic liquids, which will be a topic of the further discussion.

#### Applications of ionic liquids for the synthesis of organic carbonates

**Scheme 8:**
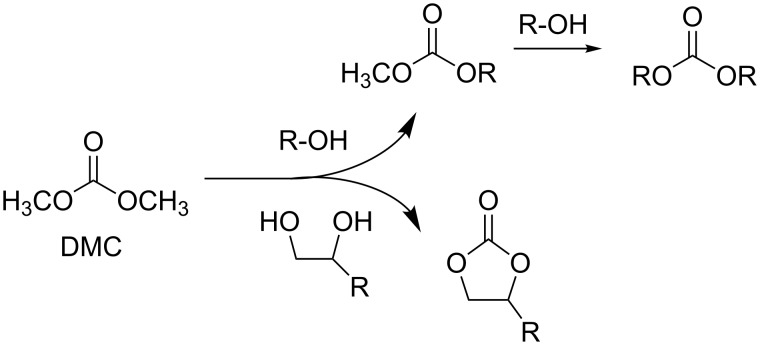
The transesterification of DMC with alcohols and diols.

The commonly used method to synthesize organic carbonates consists in the acid or base-catalyzed transesterification of dimethyl carbonate (DMC), the simplest organic carbonate, with alcohols R–OH or diols to yield either acyclic organic carbonates or cyclic carbonates, respectively (Scheme 8).

A literature survey on the synthesis of organic carbonates by ionic-liquid catalysis goes back approximately five years. Both acidic and basic ionic liquids were employed as catalysts for the pursuit of this scope. The following section is divided into two topics: the first focuses on basic catalysis, and the second on acid catalysis.

**Scheme 9:**
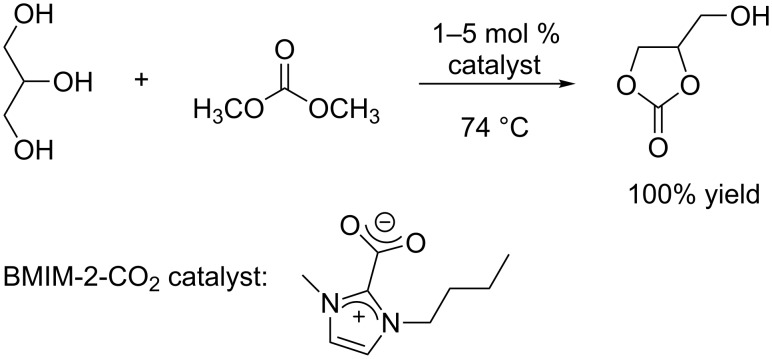
Transesterification of glycerol with DMC in the presence of 1-*n*-butyl-3-methylimidazolium-2-carboxylate (BMIM-2-CO_2_).

**Basic catalysis:** In a communication published in 2009, Naik P. U. et al. reported an expeditious protocol towards the formation of glycerol carbonate through the transesterification of glycerol with DMC (Scheme 9) [61]. They employed the ionic liquid 1-*n*-butyl-3-methylimidazolium-2-carboxylate (BMIM-2-CO_2_) in a concentration of only 1–5 mol %, and the target molecule was quantitatively obtained in 30 min at 74 °C, by using 3.2 equivalents of DMC with respect to glycerol.

**Scheme 10:**
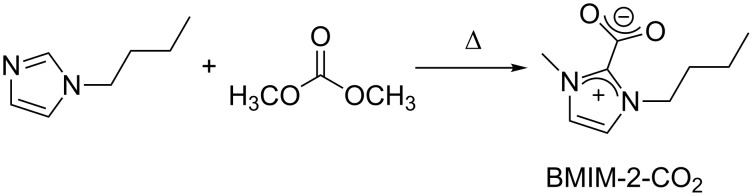
Synthesis of the BMIM-2-CO_2_ catalyst from butylimidazole and DMC.

As the BMIM-2-CO_2_ catalyst is synthesized starting from butyl-imidazole and DMC (Scheme 10), the authors also attempted to combine the in situ formation of the catalyst with the transesterification of glycerol. The process however, became much slower due to an induction time required to obtain BMIM-2-CO_2_.

The study proved that even starting from crude glycerol (which contained 41 mol % water and alkaline salts) and 5 mol % BMIM-2-CO_2_, glycerol carbonate was achieved with a 93% yield after 5 h.

More recently, Yuxuan Yi et al. described the transesterification of glycerol with DMC in the presence of four catalytic ionic liquids such as 1-methyl-3-butylimidazolium imidazolium ([BMIM]Im), 1-methyl-3-allylimidazolium imidazolium ([AMIM]Im), 1-methyl-3-butylimidazolium hydroxide ([BMIM]OH), and 1-methyl-3-allylimidazolium hydroxide ([AMIM]OH) [62]. The highest activity was achieved with [BMIM][Im]: after screening of reaction conditions, using 10 mol % of catalyst, 98.4% glycerol conversion and up to 100% selectivity towards glycerol carbonate were reached at 70 °C and ambient pressure. An easy recovery of the catalyst allowed the reuse of the IL up to three times without significant reduction of its activity.

**Scheme 11:**
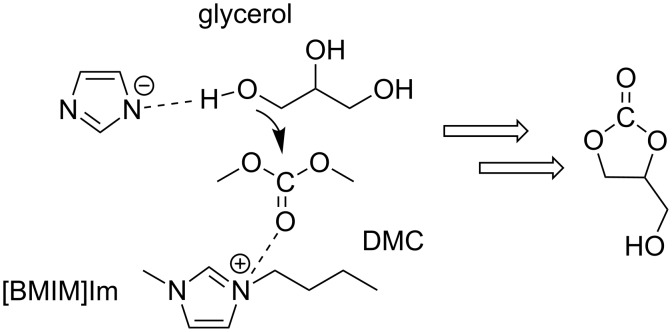
Plausible cooperative (nucleophilic–electrophilic) mechanism for the transesterification of glycerol with DMC in the presence of [BMIM]Im.

According to the authors, the result was due to a higher basicity of the imidazolium anion with respect to the hydroxy group, and to the poorer steric hindrance of the AMIM cation with respect to the BMIM cation, the latter exerting less effective interactions with the corresponding (imidazolium) anion. Regardless of its nature, the cation might also activate DMC towards nucleophilic addition: a cooperative nucleophilic–electrophilic mechanism could therefore operate (Scheme 11) [63].

**Scheme 12:**
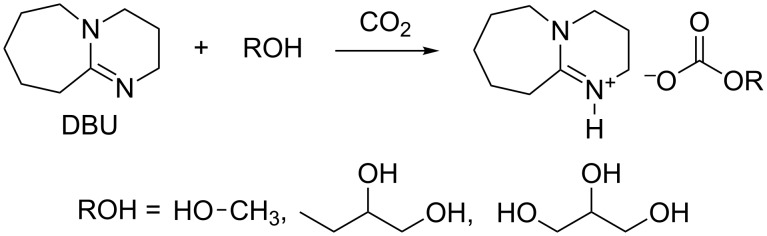
Synthesis of diazabicyclo[5.4.0]undec-7-ene-based ionic liquids.

Munshi et al. also recently investigated the reaction of glycerol and DMC by proposing a novel ionic liquid catalyst based on the reaction of diazabicyclo[5.4.0]undec-7-ene (DBU) with an alcohol ROH and CO_2_ (Scheme 12) [64].

**Scheme 13:**
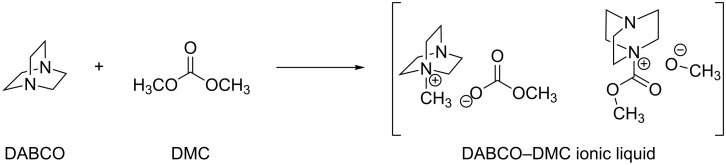
Synthesis of the DABCO–DMC ionic liquid.

As best found reaction conditions (only 0.22 mol % IL loading, glycerol to DMC molar ratio 1:3, 100 °C), conversion and selectivity (towards glycerol carbonate) were 96% and 82%, respectively, after 30 min reaction time. Glycidol (GD, 18%) was the major byproduct. In a further study by the same group, the formation of glycidol from glycerol carbonate was examined in the presence of the ionic liquid DABCO–DMC obtained in situ by reacting DABCO and DMC (Scheme 13) [65].

**Scheme 14:**
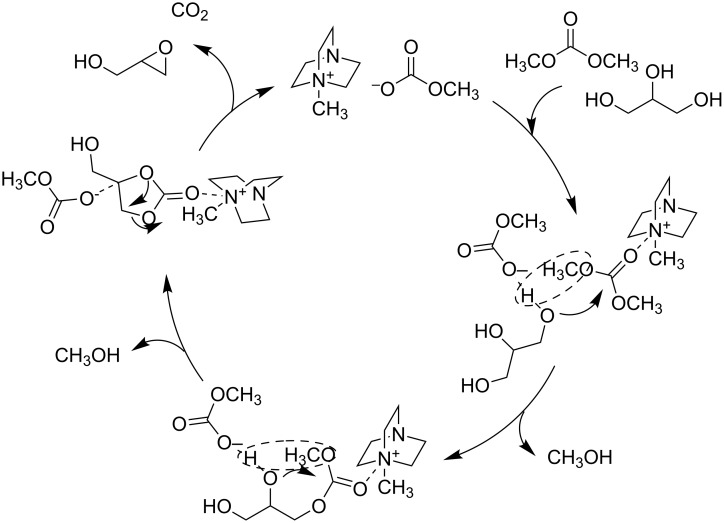
Cooperative mechanism of ionic liquid-catalyzed glycidol production.

DABCO by itself is more basic than the DABCO–DMC IL as indicated by its pH in aqueous solution. Nonetheless the DABCO–DMC IL promoted higher glycerol conversion (77% vs 19% after 10 minutes), and, most importantly, higher GD selectivity (63% compared to 45% after 30 minutes) under the same reaction conditions. In order to explain such a behavior, a cooperative mechanism for the ionic liquid catalysis was invoked, whereby the electrophilic nitrogen atom aids in activating the carbonyl moiety (Scheme 14).

It must be noted that in these two examples the selectivity of the reaction could be tuned just by changing the catalyst precursor: by switching from the DBU-based IL to the DABCO–DMC IL, the selectivity changed from 82% for glycerol carbonate, to 83% for glycidol.

In another publication, Gade et al. also achieved a high selectivity toward GD in a one-pot reaction starting from glycerol and DMC [66]. Using tetramethylammonium hydroxide ([TMA][OH]) as basic catalyst, a high selectivity (78%) for GD was reached under mild operating conditions (80 °C, 90 min). The results suggested that the decarboxylation of glycerol carbonate increased with increasing catalyst concentration in solution and thus, the high basicity of the catalyst was not the sole reason for the high activity.

**Scheme 15:**
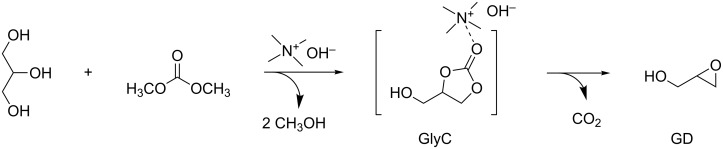
[TMA][OH]-catalyzed synthesis of glycidol (GD) from glycerol and dimethyl carbonate [46].

This implied that both, the presence of a basic (anionic) center and an electrophilic (cationic) center in the ionic liquid were involved in the reaction. It was therefore proposed that an interaction of the quaternary ammonium center with the carbonyl oxygen of glycerol carbonate (GlyC) could weaken the C=O bond (Scheme 15).

It is worth mentioning that glycidol was previously obtained at much higher temperatures (170–200 °C).

**Scheme 16:**
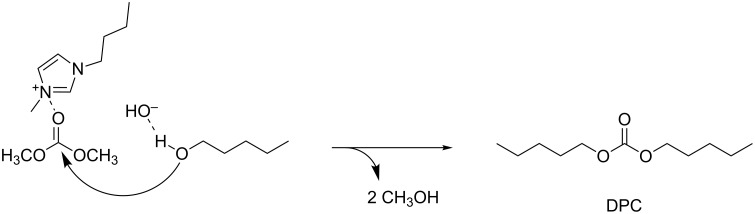
[BMIM]OH-catalyzed synthesis of DPC from DMC and 1-pentanol.

An acyclic organic carbonate that has recently received attention from the synthetic lubricant market is dipentyl carbonate (DPC). An environmentally friendly process for its synthesis has been recently proposed by the transesterification of DMC with 1-pentanol in the presence of 2 mol % of 1-butyl-3-methylimidazolium hydroxide ([BMIM]OH) as a basic ionic liquid catalyst [67]. At the best found reaction conditions (110 °C, DMC:1-pentanol in 1:4 ratio), DPC was obtained in 76% yield after 4 h reaction time. The catalyst proved to be very stable and active even after five reaction cycles where the DPC yield still exceeded 70%. The proposed reaction mechanism consisted in the activation of the carbonyl group of DMC by the hydrogen-bond interaction with the cation of the IL catalyst followed by a nucleophilic attack of 1-pentanol (Scheme 16).

**Figure 3:**
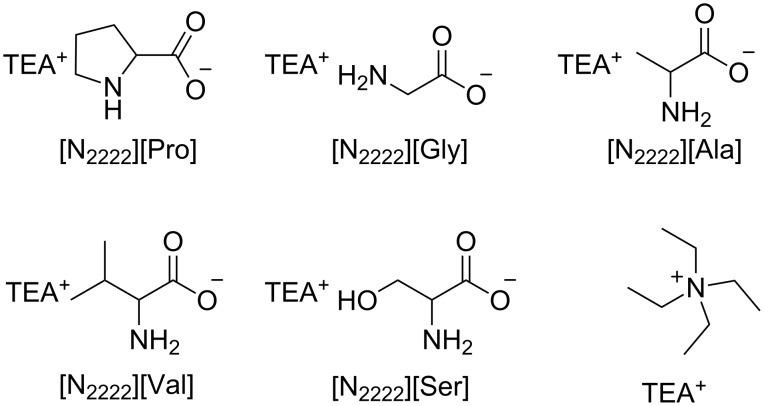
Representative examples of ionic liquids for biodiesel production.

A similar transesterification reaction of dimethyl carbonate with *n*-butanol has been accomplished using tetraethylammonium-based amino acid ionic liquids ([N_2222_][AA]) as homogeneous catalysts (Figure 3) [68]. [N_2222_][Pro] exhibited the best catalytic activity yielding an overall 72% yield of the dibutyl carbonate (DBC) product. Quantum-mechanical calculations indicated that the catalyst synergistically activated both BuOH and DMC.

**Figure 4:**
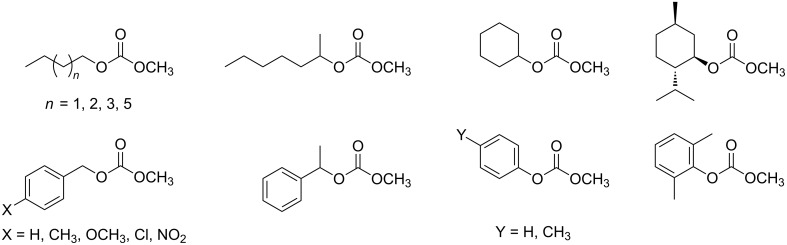
Acyclic non-symmetrical organic carbonates synthetized with 1-(trimethoxysilyl)propyl-3-methylimidazolium chloride as the catalyst.

A wide variety of acyclic non-symmetrical organic carbonates of general formula ROC(O)OCH_3_ were prepared by Kumar et al. through the transesterification of DMC using the ionic liquid 1-(trimethoxysilyl)propyl-3-methylimidazolium chloride as the catalyst (Figure 4). With a 10 mol % ionic liquid loading, the transesterification reaction of DMC with eighteen different alcohols ROH yielded the desired unsymmetrical carbonates (Figure 4) under mild reaction conditions (80 °C, DMC:ROH in 1:1 ratio) [69].

**Acid catalysis:** In the transesterification reaction of dimethyl carbonate with phenol to methyl phenyl carbonate (MPC) and diphenyl carbonate (DPhC), Deshmukh et al. studied dibutyltin oxide as a catalyst in conjunction with Brønsted and Lewis acidic ionic liquids [70]. The authors investigated the relative Lewis and Brønsted acidity of the ionic liquids by monitoring the IR bands in the presence of pyridine as a probe molecule. The highest conversions (30–39%) of phenol and the best selectivity toward DPhC were achieved using *N*-methyl-2-pyrrolidone hydrogen sulfate [NMP][HSO_4_] and choline chloride zinc chloride ([ChCl][ZnCl_2_]). The ionic liquid increases the catalytic activity of dibutyltin oxide fourfold probably by forming a highly active tin species where the anion of the ionic liquid acts as a ligand. The developed protocol was further studied for various substituted phenols, proving that electron-donating groups (EDG) at the para position enhance the substrate conversion, while electron-withdrawing groups (EWG) provide the aryl methyl carbonate with a very low conversion. Any substituents in the ortho position led to lower conversions due to an increase of the steric hindrance.

**Ionic liquid catalyzed transesterification for dimethyl carbonate production:** Ionic liquid-based catalysts brought about a number of improvements for the synthesis of DMC. As mentioned above, the synthesis of DMC through CO_2_ insertion into an epoxide and the subsequent transesterification of the formed cyclic carbonate with methanol represent a valid alternative for the industrial production of DMC [59]. Although ionic liquids can catalyze both reactions, this review will only briefly discuss the second transesterification step. Yang et al. tested many basic ILs derived from DABCO for the synthesis of DMC starting from ethylene carbonate (EC) and methanol [71]. In their study, the best performing one was 1-butyl-4-azo-1-azoniabicyclo[2.2.2]octane hydroxide ([C_4_DABCO]OH), that achieved 90% conversion, 81% DMC yield and 82% EC yield under optimized conditions (EC:methanol in a 1:10 molar ratio, 1 mol % catalyst loading with respect to EC; 4 h, 70 °C). The catalyst reusability was tested in four successive runs, in which the conversion decreased from 90 to 88% and the DMC yield from 81 to 79%, thereby proving the high stability of the investigated IL and the greenness of the process.

**Scheme 17:**
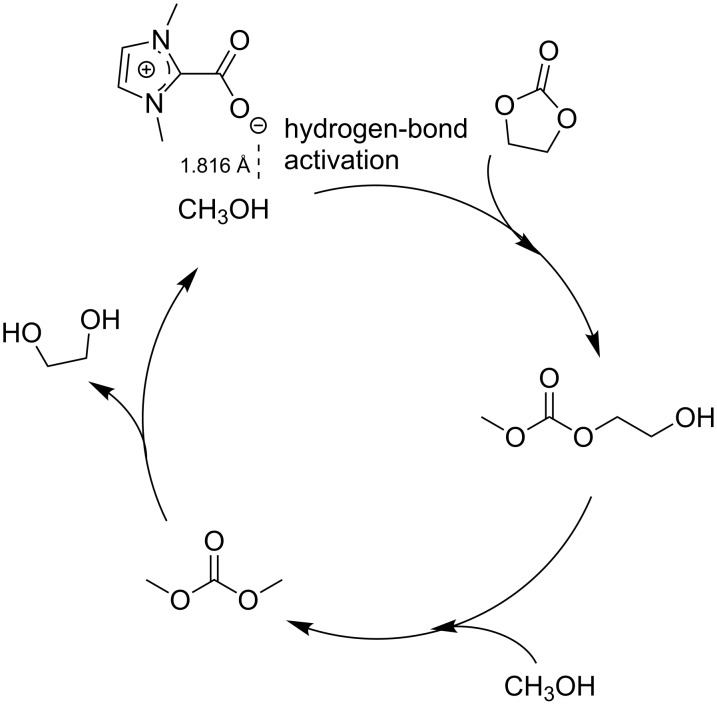
A simplified reaction mechanism for DMC production.

A one-step synthesis of DMC from ethylene oxide (EO), CO_2_ and methanol was proposed by Li et al., using a series of quaternary ammonium ILs in reactions carried out in an autoclave at 150 °C, and under CO_2_ pressure (2 MPa) [72]. Even though conversions were good after 8 h, the selectivity toward the desired product was still subject to improvement. Up to 99% EO conversion and 74% DMC selectivity were the best performances, obtained using 6-(*N’,N’*-dimethylamino)-1-(*N,N,N*-trimethylammonium)hexane iodide [N_111,6_N_11_]I as the catalyst. The reusability of the catalyst was further studied in eight subsequent reactions. Wang et al. investigated the dependence of the catalytic activity on the structure of IL cations and anions for the synthesis of DMC through the transesterification of EC with methanol [73]. They achieved the best results using a halogen and metal-free IL such as 1,3-dimethylimidazolium-2-carboxylate (DMIM-2-CO_2_), which was easily prepared by the reaction methylimidazole and DMC. Under the best found reaction conditions (1 mol % catalyst loading with respect to EC, EC:MeOH in 1:10 molar ratio, 110 °C, 80 min) the IL catalyst demonstrated high activity, as it gave 82% and 99% yield and selectivity, respectively, on DMC. Scheme 17 summarizes the reaction mechanism proposed for the synthesis of DMC. The same paper described also the results obtained by supporting the imidazolium salt onto a polystyrene resin (PS). This catalytic system proved to be highly stable and no loss of activity was detected after 200 h of reaction performed in a fixed bed reactor at 110 °C. The authors indicated the perspective of full industrial application for such a system.

#### Phosphonium salts as catalysts for the selective transesterification of carbonates

As mentioned above, the transesterification reaction between organic carbonates and alcohols or diols can be carried out in the presence of basic (e.g., tertiary phosphines and amines, alkali metal hydroxides, alkoxides, halides, carbonates, alkali metal exchanged faujasites and hydrotalcites) or acidic catalysts or co-catalysts, and under thermal (non-catalytic) conditions. All applicable catalysts show common issues: the reactions (i) often proceed beyond the mono-transesteriﬁcation products to yield the symmetrical higher organic carbonate, (ii) with polyols, primary and secondary OH groups are not discriminated leading to mixtures of different carbonates.

**Scheme 18:**
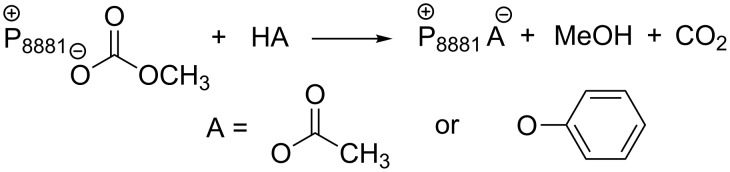
[P_8881_][MeOCO_2_] metathesis with acetic acid and phenol.

An effective strategy to improve the mono-transesterification selectivity of such reactions is through the design of new ionic liquid catalysts, such as the recently developed methyl trioctylphosphonium methyl carbonate ([P_8881_][MeOCO_2_]) and its anion metathesis analogues (Scheme 3) [34]. Of note, the preparation of these organocatalysts offers several practical advantages: (i) the synthesis of [P_8881_][CH_3_OCO_2_] involves a halide-free methylation of a trialkyl phosphine with nontoxic DMC, (ii) acetate and phenolate salt derivatives could be obtained from [P_8881_][CH_3_OCO_2_] through a chlorine-free metathesis with acetic acid and phenol (Scheme 18), and (iii) all the ILs are produced in very high purity, they are stable for months and usable straight from the reaction vessel.

**Figure 5:**
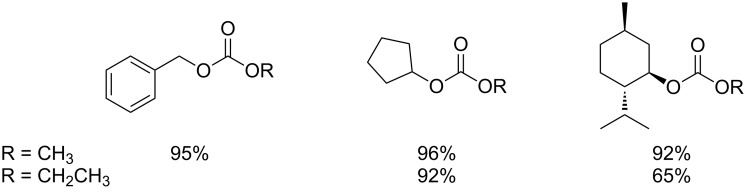
Examples of carbonates obtained through transesterification using phosphonium salts as catalysts.

Carbonate, acetate and phenolate phosphonium catalysts were shown to be effective for the mono-transesterification reaction of DMC and DEC with a number of alcohols such as benzyl alcohol, cyclopentanol and menthol [74]. Figure 5 shows some examples of the carbonates obtained in the study. The desired products were achieved at temperatures between 90 and 220 °C.

These results highlight the excellent activity and selectivity of these catalytic systems (conversion >99% and yield >90%) with respect to conventional organic and inorganic bases. In addition, the reactions proceed without decarboxylation even at high temperatures (*T* > 150 °C), as opposed to the outcome using both solid bases and zeolites, that generate large amounts of CO_2_.

**Scheme 19:**
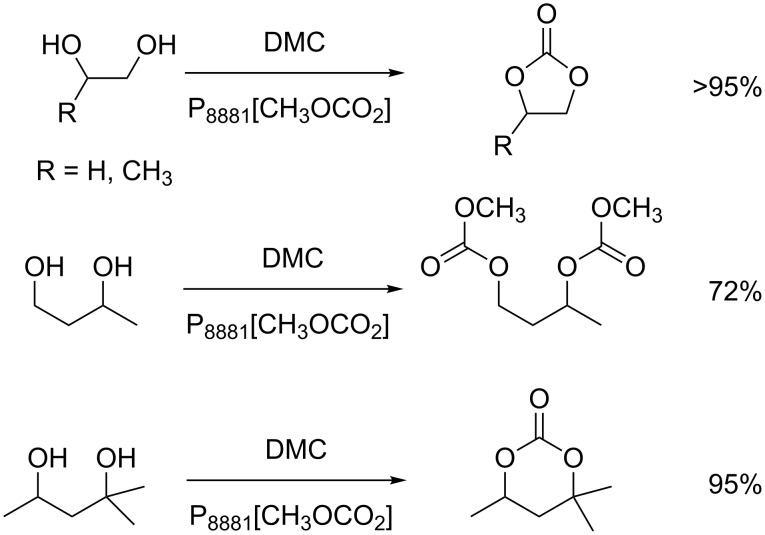
Examples of carbonates obtained from different bio-based diols using [P_8881_][CH_3_OCO_2_] as catalyst.

The transesterification activity of [P_8881_][CH_3_OCO_2_]-based ionic liquids was also tested on bio-based diols possessing primary and secondary hydroxy groups. Although a number of different products is expectable, the organocatalysts allowed highly selective reactions. For example, 1,2-diols afforded exclusively the corresponding cyclic carbonates, while 1,3-diols, depending on their structures, could yield both, cyclic or acyclic carbonates, such as the ones shown in Scheme 19 [75].

**Scheme 20:**
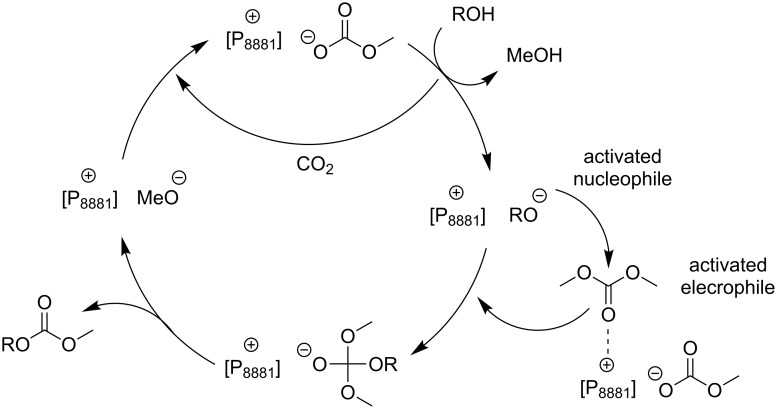
Ambiphilic catalysis for transesterification reactions in the presence of carbonate phosphonium salts, the model case of methyltrioctylphosphonium methyl carbonate [P_8881_][MeOCO_2_].

There is no direct relation of the performance of these IL-catalysts to their basicity. Curiously, it should be noted that the activity of such systems was found to be higher than that of strong bases including DBU or DABCO. This phenomenon was observed by several authors [64,76] and explained by a cooperative ambiphilic (nucleophilic–electrophilic) activation effect in which the IL anion and cation may activate respectively the nucleophile and the electrophile. Scheme 20 shows the proposed mechanisms for the exemplar transesterification of a generic alcohol ROH with DMC using [P_8881_][MeOCO_2_] as catalyst.

The catalytic cooperative activation also explains the selective formation of cyclic or linear products of Scheme 20, without the concurrent production of polycarbonate byproducts. In fact, the selectivity is plausibly due to the steric hindrance of the products, which are much less prone to electrophilic activation (by the catalyst) than the starting DMC or DEC.

Recently, phosphonium salts have been reported as transesterification catalysts of light organic carbonates (dimethyl and diethyl carbonate) with complex polyalcohols, such as cellulose. In particular, trioctylphosphonium acetate ([P_8881_][OAc]) was active for the synthesis of cellulose dialkyl carbonates which find applications as intermediates, supports for the delivery of therapeutics, imaging agents and packaging films and coatings [77].

## Conclusion

The literature survey illustrated in this review highlights three main facts. Firstly, organocatalysis by ionic liquids can be an efficient tool for base and even acid-catalyzed transesterification reactions in place of traditional inorganic or solid acids and bases. Advantages in this case are mainly the recovery and recyclability of the catalyst system and the improved selectivity that is often achievable. Secondly, the here presented reactions have a common mechanistic feature based on the cooperative nucleophilic–electrophilic catalysis by the ionic liquid. This type of ambiphilic catalysis is characterized by the nucleophile and the electrophile both being activated respectively by the anion and by the cation of the ionic liquid. Thirdly, organic carbonates – used as feedstocks or produced by transesterification – are valuable synthetic targets in view of the development of new greener solvents, additives, reagents, and in general of chemical products with improved safety and chemical properties.
